# Fabrication of Al/Mg/Al Composites via Accumulative Roll Bonding and Their Mechanical Properties

**DOI:** 10.3390/ma9110951

**Published:** 2016-11-23

**Authors:** Jinfeng Nie, Mingxing Liu, Fang Wang, Yonghao Zhao, Yusheng Li, Yang Cao, Yuntian Zhu

**Affiliations:** 1 Nano Structural Materials Center, School of Materials Science and Engineering, Nanjing University of Science and Technology, Nanjing 210094, Jiangsu, China; niejinfeng@njust.edu.cn (J.N.); lmxshenlan@163.com (M.L.); xiaofengn@163.com (F.W.); liyusheng@njust.edu.cn (Y.L.); cao_yang_leo@hotmail.com (Y.C.); ytzhu@ncsu.edu (Y.Z.); 2 Department of Materials Science and Engineering, North Carolina State University, Raleigh, NC 27695, USA

**Keywords:** accumulative roll bonding (ARB), Al/Mg alloy multilayered composite, AZ31 magnesium alloy, mechanical properties, microstructures, intermetallic compound

## Abstract

Al(1060)/Mg(AZ31)/Al(1060) multilayered composite was successfully produced using an accumulative roll bonding (ARB) process for up to four cycles at an elevated temperature (400 °C). The microstructure evolution of the composites and the bonding characteristics at the interfaces between Al and Mg layers with increasing ARB cycles were characterized through optical microscopy, field emission scanning electron microscopy (FESEM) and transmission electron microscopy (TEM). It was found that the grains of Al and Mg layers were significantly refined and Al_3_Mg_2_ and Al_12_ Mg_17_ intermetallic compound layers formed at the Al/Mg bonding interfaces. The strength increased gradually and the ultimate tensile strength (UTS) reached a maximum value of about 240 MPa at the third pass. Furthermore, the strengthening mechanism of the composite was analyzed based on the fracture morphologies.

## 1. Introduction

With the development of modern technology, a single material hardly satisfies the increasing demands for improved properties. Recently, metallic multilayered composites with two or more different components have been developed and have received a significant amount of attention due to their prominent characteristics such as excellent mechanical, electrical, magnetic properties [1,2,3]. A number of techniques have been developed to prepare these multilayered composites, such as magnetic sputtering, vapor deposition, explosive welding and friction-stir welding [4,5]. However, most of the above methods require complex processes and expensive equipment, which has limited their applications at industrial scales. As a type of severe plastic deformation combining the same or different metals, accumulative roll bonding (ARB), proposed by Saito et al., exhibits some advantages in fabricating multilayered composites such as low cost, large scale, increased strength and structural refinement [6,7,8,9]. The ARB process consisted of multiple cycles and each cycle includes rolling, cutting, stacking and solid-state deformation bonding, so that a large strain can be accumulated in the metallic sheet during the process without any geometrical change in the sheet. The rolling in ARB is not only a deformation process but also a bonding process (roll-bonding) that results in a single-body solid material [10]. Furthermore, it can be easily integrated/adapted into existing industrial rolling trains without major modifications and can be scaled up to produced sheet materials with an ultra-fine-grained (UFG) microstructure on an industrial scale [11].

Besides the conventional route for producing single component materials by ARB processing [8], recent research on ARB is focusing on the processing of multicomponent materials with the goal of producing tailored materials with properties adjusted to special needs [12]. At present, ARB process has been successfully applied to produce multilayer sheet materials, including Al/Al, Al/IF steel, Al/Cu, Cu/Zn and Al/SiC_p_ composites [13,14,15,16,17,18,19]. All of these metals possess excellent ductility so that they can continuously maintain more compatible deformation until high cycles without necking. Although a variety of alloys can be used to prepare multilayer materials, Mg alloy and Al alloy have received wide attention, and they have great potential in automotive industry because of their prominent combination of high specific strength from Mg alloy and excellent corrosion resistance from Al alloy [20,21]. Furthermore, as lightweight structural metals, the combination of Mg alloy and Al alloy could reduce the carbon dioxide emissions by decreasing the weight of the vehicles in the automobile industry. Al/Mg/Al multilayered composite with Mg in the center coated by Al not only improves the corrosion resistance property by isolating Mg but also produces compressive stress on the surface to strengthen its deformation ability. However, Al and Mg have different crystal structures (fcc and hcp) and plastic instability will occur during the co-deformation. Furthermore, Mg alloy sheets develop a strong texture during the rolling process, and the basal planes orient themselves in the rolling plane. The texture combined with the lack of a sufficient slip system leads to low formability at low temperatures [22,23,24]. Only at a higher temperature above 280~300 °C and moderate strain rates can the pyramidal <c+a> system be cativated, and then Mg alloys can exhibit improved ductility and be readily formable [22]. Therefore, the limited ductility of most Mg alloys has made it difficult to fabricate Al/Mg composite plates at room temperature. The inconsistent deformation of Al and Mg during the ARB process may lead to the necking of the Mg layer after high cycles and then hinder the close bonding of the composites. Thus, it is necessary to increase the rolling temperature to fabricate the Al/Mg multilayered composites via the ARB process.

In the recent research, the Al/Mg multilayered composites were formed by hot rolling at an elevated temperature above 400 °C [25,26]. Most of them focus on the study of the Al/Mg/Al three-layered composite and the microstructure evolution at the interfaces. In the present research, a series of multilayered Al/Mg/Al composites up to 24 layers were fabricated by the ARB process with a preheating procedure at 400 °C. Furthermore, the grain size variation of the Al and Mg alloy and the microstructure evolution of the intermetallic layer formed at the interface during the ARB process were investigated in detail. The mechanical properties of the composites in different cycles were also studied.

## 2. Experimental Materials and Procedures

The raw materials used in this study were commercial pure Al 1060 and AZ31 magnesium alloy with the composition listed in Table 1. The pure Al alloy and AZ31 alloy were homogeneously annealed in N_2_ atmosphere at 400 °C for 2 h. The initial sizes of the Al alloy and Mg alloy plates were 180 × 40 × 1.5 mm^3^ and 180 × 40 × 1 mm^3^, respectively. Thus the total thickness of the primary sandwich structure of Al/Mg/Al was 4 mm.

**Figure 1:**
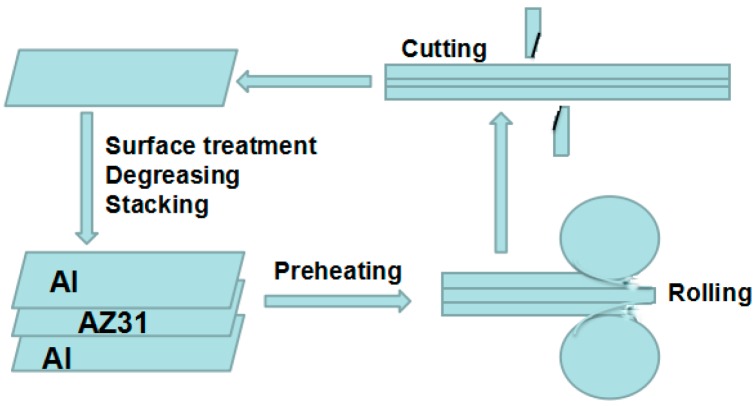
Diagrammatic illustration of the accumulate roll-bonding (ARB) process.

Figure 1 illustrates the principle and procedure of the ARB processing. Firstly, the three Al/Mg/Al sheets were stacked and fastened after degreasing by acetone and sanding by wire brush in order to eliminate grease and oxide film. After being preheated at 400 °C for 5 min in a furnace, they were roll-bonded immediately with a reduction ratio of 50% in thickness in a single cycle. The roll-bonded sheet was cut into two halves and stacked again, and this pre-treatment process was repeated for another three times (the second, third, and fourth cycles). The whole process was conducted without lubricant to produce a large shear strain. All ARB processes were carried out on a 120 mm diameter roll-mill at a rolling speed of 0.34 m/s.

The microstructures of the composites were characterized using a scanning electron microscope (SEM, Quanta 250F, FEI, Hillsboro, OR, USA) equipped with Oxford energy dispersive X-ray spectrometer (EDS) and Electron Backscattered Diffraction (EBSD). The grain structure of the Mg alloy was observed using an optical microscope (OM, Axio Vert A1, ZEISS, Oberkochen, Germany) in the plane of RD-ND. Vickers hardness experiment was carried out on the RD-ND plane of the multilayered composite using a tester (HMV-G 21DT, SHIMADZU, Tokyo, Japan) at the load of 0.49 N holding for 15 s. For each specimen, at least seven points were tested to obtain a mean value with a standard deviation error. Tensile tests were conducted using a constant speed of 0.5 mm/min, corresponding to a strain rate of 5.6 × 10^−4^ s^−1^ on the Walter+bai LFM 20 KN universal test machine. Then tensile test specimens were machined from the ARB processed sheets oriented along the rolling direction according to the ASTM: E8M standard. The gauge width and length of the tensile specimens were 2 and 15 mm, respectively, and their length was 45 mm. To get accurate results, at least five tensile experiments were conducted on each sample and subsequently averaged.

## 3. Results and Discussion

### 3.1. Interface Microstructure of the Al/Mg/Al Multilayer Composites

**Figure 2:**
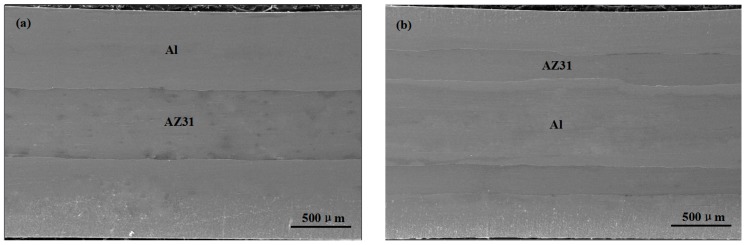

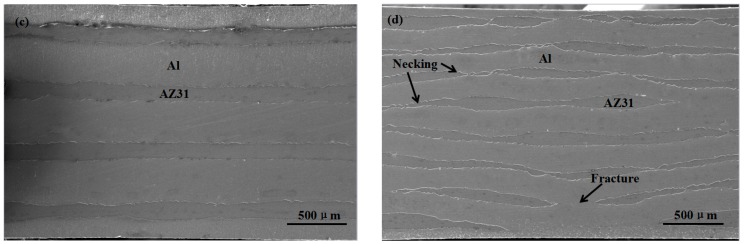
SEM micrographs of ARBed Al/Mg composites: (**a**) primary sandwich; (**b**) 2nd; (**c**) 3rd; (**d**) 4th cycle.

Figure 2 shows the microstructure variation of the Al/Mg/Al composites on the RD-ND plane during the different ARB cycles. No obvious voids and gaps were observed at the straight bonding interface of Al/Mg as shown in Figure 2a, indicating that Al and Mg layers bonded together after the first cycle at the present rolling conditions. Figure 2b shows the microstructure of composites with six layers after two ARBed cycles, but the new formed Al/Al interface cannot be seen, clearly demonstrating that the two parts were also successfully bonded together. Similar phenomena were also found in the samples after the third cycle as shown in Figure 2c. Meanwhile, Al/Mg interfaces remained straight in the earlier three cycles, which indicated that AZ31 alloy behaved a good ductility due to the activation of the nonbasal slip system after the preheating process and co-deformed with the pure Al alloy. However, during the fourth cycle, the Mg layers begin to neck and fracture locally as shown by the arrows in Figure 2d.

**Figure 3:**
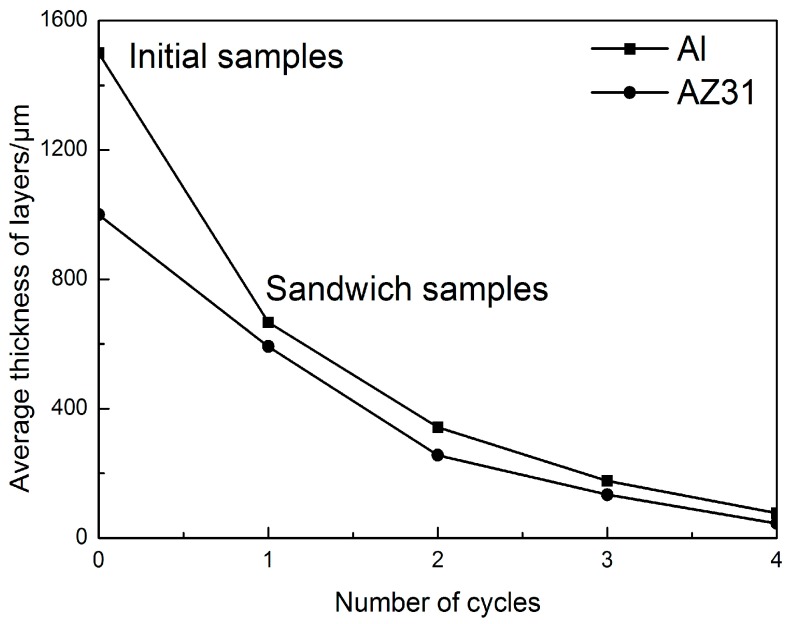
Thickness variations of Al and Mg layers in Al/Mg/Al composite during ARB cycles.

The variations of the average thickness of Al and Mg layers versus the ARB cycle is show in Figure 3. As the ARB proceeded and the strain is increased, the thicknesses of the Al and Mg layer are decreased. As it can be seen, Al layer is half time thicker than Mg layer at the initial stage, but they become similar after the fourth cycle.

**Figure 4:**
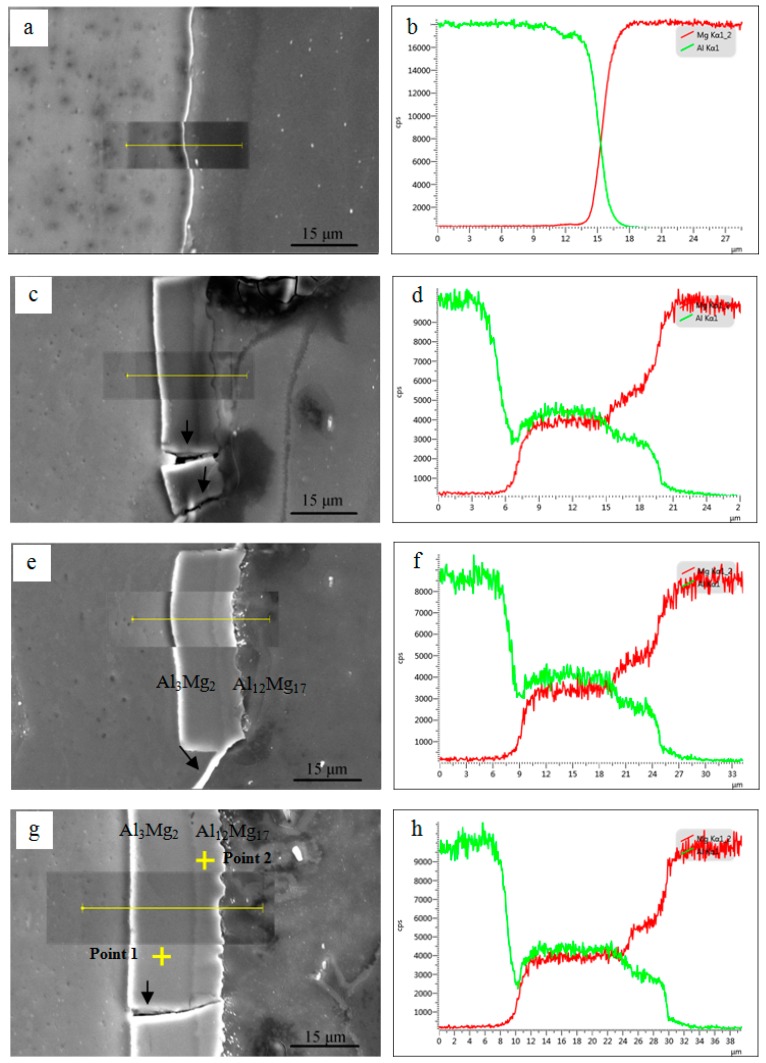
SEM images and corresponding EDS line-scanning analysis across the interfaces of the Al/Mg composite after (**a**,**b**) one; (**c**,**d**) two and (**e**,**f**) three; (**g**,**h**) four cycles.

Figure 4 shows SEM images and EDS line-scanning analysis of the interfaces of Al/Mg/Al laminated composites after one, two, three and four ARBed cycles, respectively. As shown in Figure 4a,b, an ‘X’ shape composition profile can be seen to form across the interface between the Al and Mg layers, which indicates that a certain amount of atomic diffusion occurred at a very narrow area during the first cycle. It is noticeable that a layer of intermetallic compounds about 13 μm is formed at the interface after two ARB cycles as displayed in Figure 4c,d. Furthermore, the average thickness of the intermetallic compounds layer increased to about 16 μm and 23 μm after three and four cycles, respectively, as shown in Figure 4e,g. There are gradual composition changes of Al and Mg elements across the interface shown by the component curves in Figure 4c–h, which indicates that a solid solution was formed through the atomic inter-diffusion behaviors between Al and Mg alloys.

**Figure 5:**
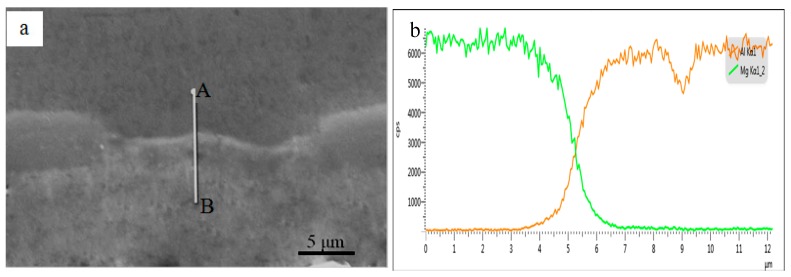
EDS line-scanning analysis of the fresh interface in the Al/Mg composites after three cycles: (**a**) SEM image; (**b**) element distribution of Mg and Al along line A-B across the interface.

Furthermore, it can be seen that the interfacial layer consists of a light shade layer next to the Al side and a gray shade layer next to the Mg side. According to the EDS point analysis as shown in Table 2, it is assumed to be Al_3_Mg_2_ next to the Al alloys and Al_12_Mg_17_ phase next to the Mg alloy, respectively [27,28]. Besides, the diffusion zone of Al_3_Mg_2_ is thicker than that of the Al_12_Mg_17_, indicating that the diffusion ability of Al atoms is lower than that of Mg atoms [29]. Due to the Al_3_Mg_2_ and Al_12_Mg_17_ being brittle phases, they are broken in the further ARBed cycle and then cracks can be observed at the interface as shown by the arrows in Figure 4c,g. However, fresh metal will squeeze into the cracks and bond together again at the interface under the rolling force shown by the arrow in Figure 4e. In addition, Figure 5 shows the interface analysis between Al and Mg layers in the laminated composites after the third cycle. It is clearly that the intermetallic compounds layer was broken and discontinuous. Figure 5b shows the element distribution of Al and Mg along the line A-B across the interface without metallic compounds. An ‘X’ shaped composition profile can be seen across the interface between Al and Mg layers, indicating that the fresh Al and Mg layer squeezed out and bonded together under the rolling force in the next cycle.

**Figure 6:**
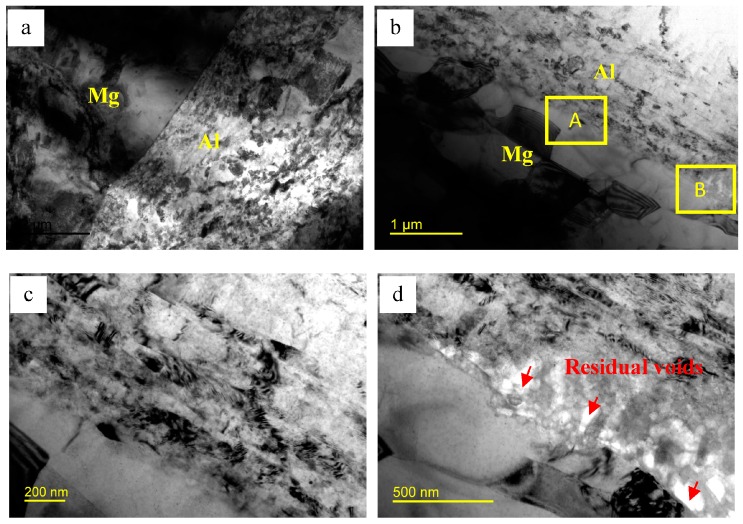
(**a**,**b**) TEM bright field images of the interface of Al/Mg/Al laminated composites after three cycles and (**c**,**d**) magnified microstructures of interface marked by A and B in (**b**).

Figure 6 shows the TEM images of the Al/Mg interface microstructure of the composites after three ARB cycles. It can be found the equiaxed grains of AZ31 layer near the interface are smaller than 1 μm, while the grains of Al layer are elongated as shown in Figure 6a,b. After a higher magnified observation at the location marked by A in Figure 6b, it is noticed that the interface bonding well with no intermetallic compounds. However, there are still some residual voids at the interface zone marked by B, as shown in Figure 6d. According to Figure 4, one can see that the brittle intermetallic compounds were broken in the subsequent rolling process and then the fresh Al and Mg layers squeezed out and bonded together again under the rolling force. Therefore, it is supposed that the fresh Al and Mg layers bond with no visible intermetallic compounds, but some residual voids can be seen, which can be eliminated by the further bonding.

### 3.2. Grain Structural Evolution of the Al/Mg/Al Multilayer Composites

**Figure 7:**
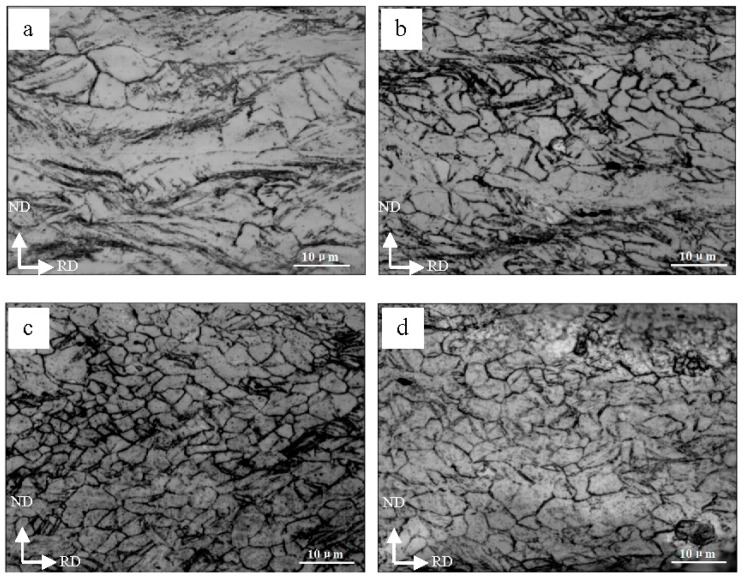
Optical micrographs showing the microstructure of Mg layer in the Al/Mg/Al laminated composites after (**a**) one; (**b**) two; (**c**) three; (**d**) four cycles.

Figure 7 shows the microstructure of Mg layer in the Al/Mg/Al laminated composites after different ARBed cycles. It can be seen that the development of grain structure of the Mg layer in the TD plane was inhomogeneous. The elongated grain structure and lots of deformation zones can be seen along the rolling direction after one cycle as shown in Figure 7a. With increased ARBed cycles, the density of dislocation also increased and then dislocation cells were formed due to the dislocation tangle as displayed in Figure 7b. Besides, it is also seen that some grains are refined significantly after two cycles due to the fragment of the elongated grains during the rolling process. After three ARBed cycles, a homogeneous microstructure with refined grains of about 2.5 μm was obtained (Figure 7c). However, the grains of the Mg layer coarsened slightly after the fourth ARBed cycles as shown in Figure 7d.

It is known that the new fine grains appeared during the roll bonding process because of dynamic recrystallization (DRX) and formed at the original grain boundaries [28,30]. The fraction of fine grains increases with the ARBed cycles because of the accumulated stress level. After three cycles, the microstructure became homogeneous and thus the DRX in the composites is completed at the third cycle in the present conditions [31]. With an increased ARBed cycle, both the higher strain energy in the samples and the elevated rolling temperature promoted grain growth [32]. Hence, the grains of Mg layer cannot be refined further.

**Figure 8:**
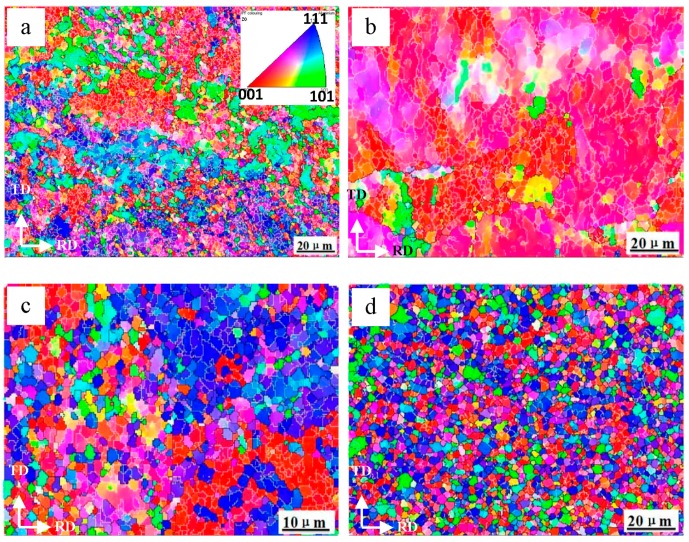
EBSD maps of surface Al layer in the Al/Mg/Al laminated composites after (**a**) one; (**b**) two; (**c**) three; (**d**) four cycles.

Figure 8 illustrates the microstructure of the surface Al layer in the Al/Mg/Al composites with different ARB cycles. It can be seen that the initial grain boundaries cannot be identified clearly after the first two cycles and a large number of low-angle grain boundaries are distributed uniformly in the large grain (Figure 8a,b). It is known that the accumulation of dislocations induced by plastic strain led to a development of the low-angel boundaries which subdivided grains into dislocation cells [33]. With an increased ARBed cycle, it can be seen that the number of high-angle boundaries increased and thus the grains are refined significantly as shown in Figure 8c, indicating that most of the initial grains were fragmented into the equiaxed fine grains under the rolling stress. After the fourth cycle, a homogeneous microstructure of the 1060 Al layer with refined grains was obtained as shown in Figure 8d and the average grain size was about 2 μm.

**Figure 9:**
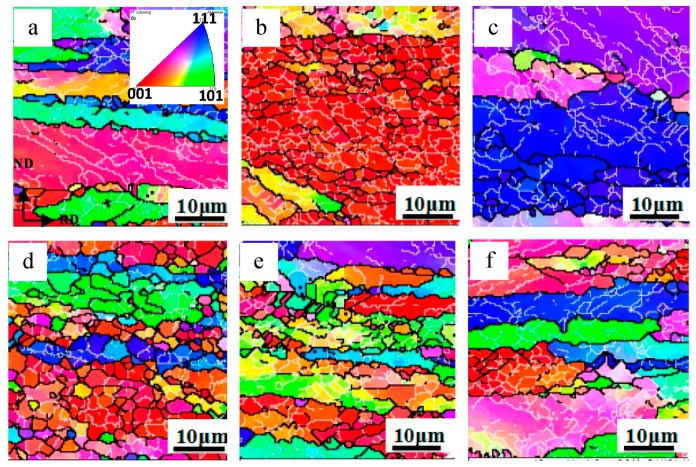

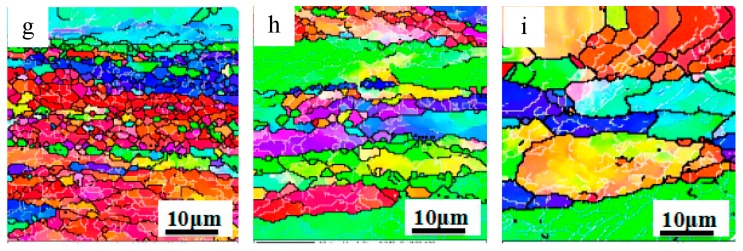
EBSD maps of Al layers through thickness of the laminated composites with different cycles: (**a**) surface Al layer after the 1st cycle; (**b**,**c**) surface and center layer after the 2nd cycles; (**d**–**f**) surface, subsurface and center layer after the 3rd cycles; (**g**–**i**) surface, subsurface and center layer after the 4th cycles.

Figure 9 shows the EBSD maps of Al layers through thickness of the Al/Mg/Al sheets with increasing ARB cycles. The elongated grains along the rolling direction were distributed with a large number of low-angle grain boundaries can be seen clearly after the first cycle. Then, with increasing strain after two cycles, high-angle boundaries developed and the grains in the surface layer are refined significantly as shown in Figure 9b. However, the grains in the center layer (Figure 9c) are much coarser than those in the surface layer. It should be noted that in ARB process half of the surface regions comes to the center in the next cycle and that procedure is repeated. After the third cycle, the grains in the surface layer are refined significantly as shown in Figure 9d. Besides, the grains in the subsurface and middle layers are shown in Figure 9e,f, respectively, and a large grain size gradient can be seen across the thickness of the sheets. With increasing cycles, all the grains through the thickness are refined further by the increased strain and the grain size gradient is increased as well as shown in Figure 9g–i. After the fourth cycle, the average grain size in the surface layer is 2 μm, while that in the center layer is 10 μm.

### 3.3. Mechanical Properties of the Al/Mg/Al Composites

**Figure 10:**
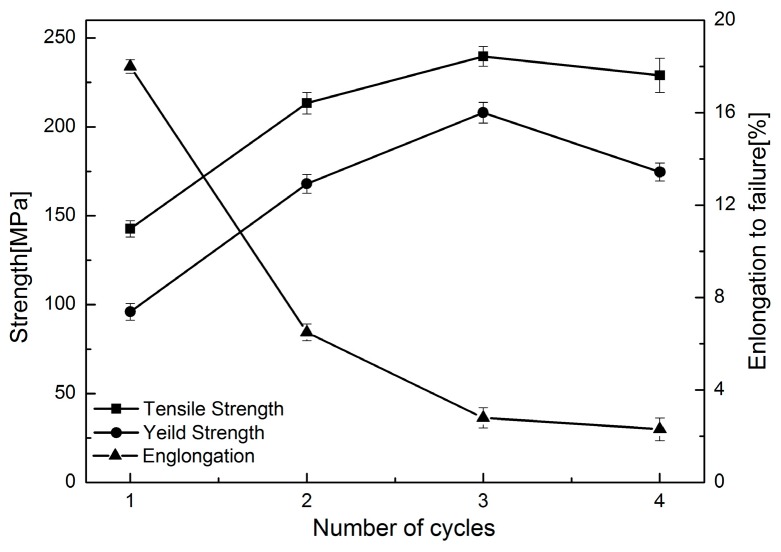
Mechanical properties of Al/Mg/Al multilayered composites with different ARB cycles.

The tensile properties of Al/Mg/Al multilayered composite were measured along the rolling direction and Figure 10 shows the variation in tensile properties with the ARBed cycles for the composites. It can be seen that both the ultimate tensile strength and yield strength increased to the maximum value at the third cycle and then decreased with increased rolling cycle. The maximum yield strength and tensile strength reached 178 MPa and 240 MPa, respectively. With regards to elongation, it shows a dramatic decrease after two ARBed cycles, and then a slight decrease with increasing cycles. It is noted that the ultimate tensile strength of the sandwich composite reached 140 MPa, which is slightly higher than the sum of the strength of separate layers, as calculated using the role of mixture ($\sigma =0.75\sigma_{Al}+0.25\sigma_{Mg}=133$ MPa,). $\sigma_{Al}$ and $\sigma_{Mg}$ are the ultimate tensile strengths of the starting materials used in the experiment, and their values are 106 and 213 MPa, separately.

**Figure 11:**
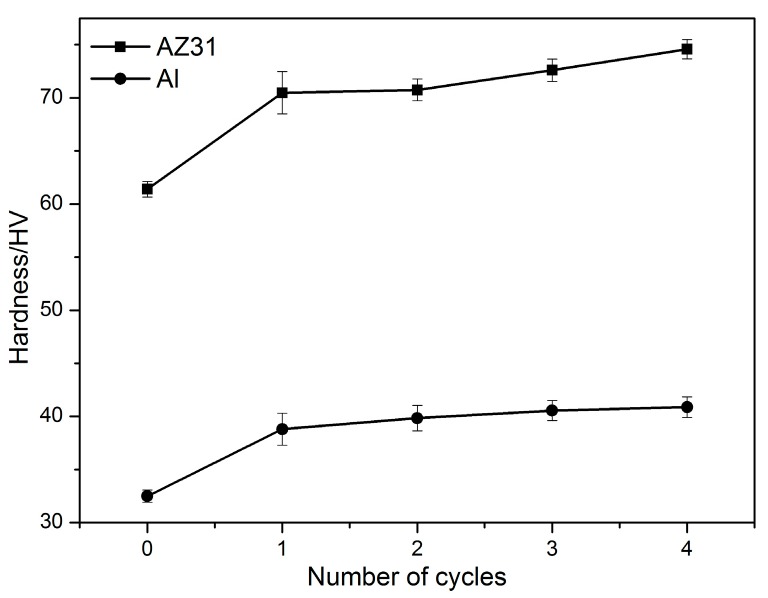
Hardness variation of Al and Mg layers in the Al/Mg/Al composites at different ARB cycles.

Furthermore, the hardness of Al and Mg layer in the Al/Mg/Al multilayered composites with different ARBed cycles were also measured and shown in Figure 11. It can be seen that both the hardness of Al and Mg layers increased rapidly after the first cycle because the deformation induced a large amount of dislocation for work hardening. However, the hardness of Al and Mg layers increased slowly with further increased rolling cycles.

**Figure 12:**
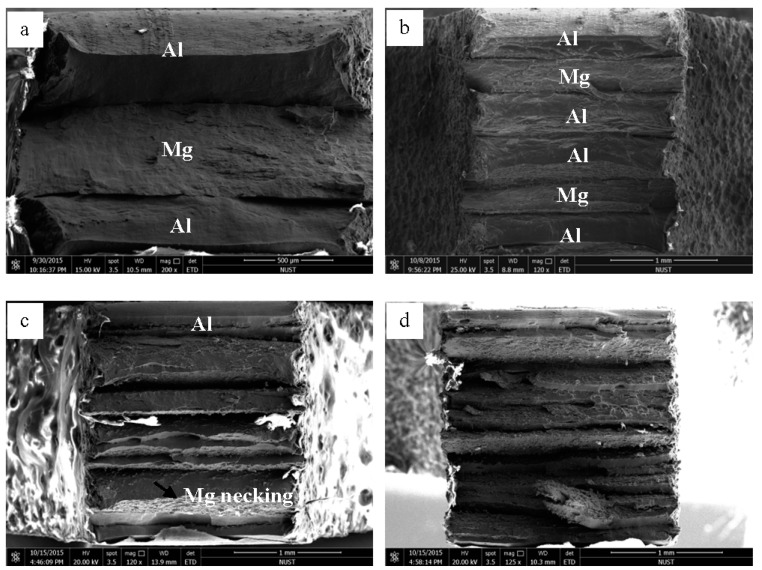
The panoramic SEM fractographs of the Al/Mg/Al laminated composites fabricated after (**a**) one; (**b**) two; (**c**) three and (**d**) four cycles.

Figure 12 shows the fracture morphologies of the Al/Mg/Al laminated composites fabricated by different ARBed cycles after tensile tests. It can be seen that the laminated composite with one ARBed cycle was delaminated and deformed independently until fracture due to the weak bonding force between the Al and Mg layers as shown in Figure 12a. After two and three cycles, the composites deformed as a whole structure with a trace of delamination in the fracture surface as shown by Figure 12b,c, indicating that the laminated composite possessed a higher bonding force [28]. However, after the fourth cycle, the bonding strength was decreased by the fragile intermetallic compounds as shown in Figure 12d.

### 3.4. Discussion

It is shown in Figure 2 that the Mg layers were elongated during the first three cycles and preserved their coherency in most of the regions. After the fourth cycle, necking and fracture of the Mg layer took place through the samples (Figure 2d). Generally, during the plastic co-deformation of dissimilar metals, instabilities were due to the differences in mechanical properties of (Al and Mg) causing necking and fracture in hard layers [34,35]. It should be mentioned that difference in mechanical properties of two dissimilar layers of Al/Mg composites leads to non-homogeneous fragmentation of Mg layer. It is noticed that the necking site in each Mg layer seems to be at 45° angle to the rolling direction and similar results were also reported by K. Inal et al. [36]. Based on simulation method they reported that the localized deformation in the form of a shear band passing through the center of the specimen is formed at approximately 47° to the loading direction. Min et al. also demonstrated that shear bands in the matrix around the interface of the matrix and reinforcement move inside the hard phase due to their lower formability and cause shear and separation in the hard phase [37]. Therefore, it is supposed that the shear bands formed close to the interface with increasing strain and cut the Mg layer due to its worse deformability.

As shown in Figure 3, the thickness of both the Al and Mg layers rapidly decreased in the initial ARB cycles as did deformation ability. This is also due to the formation of shear bands close to the interface during the rolling process, which has been observed by Quadir et al. [13]. The shear band formation is related to the differences in flow strength of adjacent hard and soft layers. Being exposed to the same load during rolling, the soft layer is subjected to higher strains than the harder layer, which results in an in-plane shear force at the interface. Therefore, surface Al layer was subjected to higher strains than Mg layer in the present condition. Consequently, the thickness of Al and Mg became similar with increasing ARB cycles.

Furthermore, the shear that occurred during the ARB process also influenced the grain refinement process of the layers. It is known that the surface regions had greater shear deformation because of the friction between the rolls and the sheet. Tsuji et al. had confirmed that a very large shear strain had been introduced into the surface layer of the rolled sheet and the shear strain distributed in the center layer was very small [38,39]. Prakash et al. also investigated the evolving texture during ARB processing by simulation and found that a through thickness gradient of texture and anisotropy developed after both rolling passes [40]. The positions showing the minimum in grain size obviously corresponded to those with maximum shear strain. In the ARB processed materials, the surface layer came to the center and had plain strain deformation in the next cycle. Thus, the shear strain distribution played an important role in the ultrgrain refinement in the ARB. It is supposed that the surface Al layer with the maximum shear strain had the minimum grain size. Besides, the surface layer (Figure 9d) came to the center in the next cycle (Figure 9i) and then the grain size increased significantly, which indicated that the refined grains coalesced and grew rapidly due to elevated temperature in the interval heating process.

The results of mechanical properties in Figure 10 and Figure 11 show that strength and microhardness increased considerably within the first three cycles of the ARB process. It is known that both strain hardening through dislocation tangle and grain refinement are the two main strengthening mechanisms in the early stage of the ARB process [15,41,42]. In the first two ARBed cycles, work hardening leads to an increase in strength and a quick decrease of the ductility for the composites. With increased cycles, grain refinement increases the strength of the composites further, while the elongation decreases slowly. However, on the other hand, the formed brittle intermetallic compounds at the Al/Mg interfaces with increased cycles at the elevated temperature also decreased the strength and elongation gradually. Especially, after the third cycle, the later one played the leading role and then the strength and elongation decreased.

It was observed that microhardness of Al and Mg layer considerably increased during ARB cycles. It exhibited a high increasing rate in early cycles of ARB while increased at a lower rate for the last cycles. On one hand, it is supposed that parts of dislocation were recovered in the subsequent preheating process and thus the hardness could not increase rapidly in the further ARBed cycle. On other hand, the hardness of the Al alloy could not rise due to the occurrence of recrystallization [42]. Additionally, the repeated ARB process causes more homogeneous strain distribution through the multilayered composite thickness, thus the hardness of the surface and center layers become to be uniform [43]. Results also showed that grain refinements contributed less to the increase of microhardness than the work hardening mechanism.

The rupture mode in Al/Mg/Al composites has been shown in Figure 12. It can be seen that the Mg layers in the first two samples exhibited ductile necking before fracture, which may be the main reason for the high elongation of the composites. However, a thick layer of fragile intermetallic compounds formed at the interface in the composites during the fourth cycles, which decreased the bonding strength and cracked severely in the tensile test as shown in Figure 12d. Therefore, it can be deduced that the intermetallic compound cracking is the main reason for the large strength and elongation decrements along rolling direction after three cycles.

## 4. Conclusions

(1) A kind of Al/Mg/Al multilayered composite was successfully produced using 1060Al and AZ31 plates via an accumulative roll bonding (ARB) process, up to 4 cycles at an elevated temperature (400 °C) in this work.
(2) The Al and Mg layers remained straight till the third ARBed cycle, indicated that the AZ31 alloy exhibited a good ductility under the preheat temperature and co-deformed with the pure Al alloy in the rolling process. However, two layers of intermetallic compounds, Al_3_Mg_2_ and Al_12_ Mg_17_, formed at the bonding interfaces due to the elevated rolling temperature after two cycles. However, they were broken in the subsequent cycle and the fresh Al and Mg layer bonded together again.
(3) The grains of Al and Mg layers in the composites were also refined significantly and a homogeneous microstructure was obtained after three ARBed cycles. The average grain sizes of the refined Al and Mg alloys were 2 μm and 2.5 μm, separately.
(4) The UTS and YS of the composites increased to a maximum value at the third cycle and then decreased with a further cycle. The maximum YS and UTS reached 178 MPa and 240 MPa, respectively. The EL shows a similar rule for strength. Based on the fracture morphologies analysis, it is supposed that the intermetallic compound cracking was the main reason for the strength and elongation decrements with increased rolling cycles.

## Figures and Tables

**Table 1:** Chemical composition of AZ31 used in the experiment.

Element	Al	Zn	Mn	Si	Cu	Fe	Ni	Mg
Content (wt %)	2.94	0.9	0.23	0.01	0.01	0.003	0.00053	Bal.

**Table 2:** EDS point analysis of the interfacial layer.

Element	Al K		Mg K	
	Weight %	Atomic %	Weight %	Atomic %
Point 1	47.6	61.1	52.4	38.9
Point 2	40.8	38.3	59.2	61.7
